# Postoperative kinesiophobia in patients undergoing video-assisted thoracoscopic lung surgery: a longitudinal study

**DOI:** 10.3389/fsurg.2026.1874007

**Published:** 2026-07-23

**Authors:** Min Zhang, Xiaoshuang Dan, Qianqian Chen, Zhengmei Li, Yueru Li

**Affiliations:** Department of Thoracic Surgery, The Third Affiliated Hospital of Chongqing Medical University, Chongqing, China

**Keywords:** influencing factors, kinesiophobia, longitudinal study, lung surgery, video-assisted thoracoscopic surgery

## Abstract

**Objective:**

To investigate the current status of postoperative kinesiophobia in patients undergoing video-assisted thoracoscopic surgery (VATS) and to identify its influencing factors.

**Methods:**

A longitudinal study was conducted among patients who underwent VATS lung surgery between January 2024 and August 2024. Data were collected using a general information questionnaire, the Tampa Scale for Kinesiophobia (TSK), the Visual Analogue Scale (VAS) for pain, the Postoperative Pain Self-Management Behavior Questionnaire (PPSMB), and the Self-Rating Scale of Sleep (SRSS).

**Results:**

Of the 187 enrolled patients, 101 (54.01%) developed kinesiophobia on postoperative day 1, with a median TSK score of 41.00 (interquartile range: 38.00–42.00). During follow-up until discharge, 83 patients transitioned from kinesiophobia to non-kinesiophobia, whereas 18 patients remained persistently kinesiophobic. Univariate analysis indicated that postoperative pain intensity, pain self-management ability, operation duration, chest tube dwell time, length of hospital stay, and sleep quality significantly influenced the occurrence of kinesiophobia. Multivariate logistic regression analysis revealed that moderate to severe pain, postoperative pain self-management ability, and operation duration were independently associated with kinesiophobia. Receiver operating characteristic (ROC) curve analysis showed that pain intensity, pain self-management ability, operative duration, and their combined model yielded high specificity and sensitivity in predicting kinesiophobia.

**Conclusion:**

The incidence of postoperative kinesiophobia is relatively high in patients undergoing VATS lung surgery. Healthcare providers should pay close attention to moderate-to-severe postoperative pain, patients' pain self-management behaviors, sleep status, operation duration, chest tube duration, and length of hospitalization. Given that prolonged kinesiophobia poses greater challenges to recovery, early identification, timely intervention, enhanced patient education, and guided rehabilitation training are crucial to reducing its occurrence and promoting postoperative recovery.

## 1 Introduction

In recent years, with the advancement of minimally invasive thoracic surgery and the widespread adoption of Enhanced Recovery After Surgery (ERAS) protocols, video-assisted thoracoscopic surgery (VATS) has emerged as the primary modality for treating pulmonary diseases (1). Compared with traditional thoracotomy, VATS entails less chest wall trauma and avoids significant neuromuscular injury. Nevertheless, the incidence of postoperative acute pain remains as high as 65% (2). Despite the availability of multimodal analgesic strategies such as paravertebral nerve block, intercostal nerve block, and intravenous patient-controlled analgesia, the incidence of moderate-to-severe pain within the first 48 h postoperatively can still reach 15.6% (3).

Kinesiophobia is defined as an excessive, irrational fear of physical movement or activity, arising from heightened pain sensitivity triggered by injury or persistent pain (4). Its development is closely intertwined with pain perception. Painful insults increase nociceptive sensitivity, which in turn reinforces fear-avoidance beliefs. Once kinesiophobia becomes established, patients often experience marked anxiety and depression; without appropriate management, this condition may ultimately culminate in a pathological state, diminished quality of life, and suboptimal postoperative recovery (5).

To date, research on postoperative kinesiophobia specifically in patients undergoing thoracoscopic lung surgery remains scarce in China, and standardized assessment and rehabilitation protocols for thoracic and abdominal surgical populations are still lacking (6, 7). Therefore, this study was designed to investigate the current status and influencing factors of postoperative kinesiophobia in VATS patients, with the aim of providing evidence for risk factor control, targeted intervention strategies, and postoperative rehabilitation guidance.

## 2 Materials and methods

### 2.1 General information

Using convenience sampling, 200 patients who underwent VATS lung surgery in the Department of Thoracic Surgery from January 1, 2024, to August 30, 2024, were initially enrolled. Four patients withdrew voluntarily and nine were excluded, yielding a final analytical sample of 187 patients.

Inclusion criteria: (1) primary pulmonary disease meeting surgical indications (8–10), with the patient undergoing their first VATS procedure; (2) age ≥18 years; (3) preoperative pain score of 0; (4) no history of psychiatric illness and adequate cognitive function; (5) voluntary participation and completion of the survey.

Exclusion criteria: (1) severe intraoperative or postoperative complications (e.g., active bleeding, bronchopleural fistula, severe complications involving the thyroid, heart, etc.); (2) concurrent malignancies in other systems; (3) inability to cooperate due to mental or neurological disorders; (4) hearing impairment preventing cooperation; (5) concurrent participation in other studies.

Withdrawal criteria: voluntary withdrawal from the study at any time point.

This study was approved by the Ethics Committee of The Third Affiliated Hospital of Chongqing Medical University (Approval No. 2023-01-54), and written informed consent was obtained from all participants.

### 2.2 Research methods

#### 2.2.1 General information questionnaire

The questionnaire collected demographic data [gender, age, occupation, surgical history, marital status, education level, insurance type, living arrangement, smoking history, length of hospital stay, number of hospitalizations, and body mass index (BMI)], disease-related data (diagnosis, comorbidities), and surgical information (type of VATS, resection extent, operation duration, chest tube details, postoperative pain intensity, and analgesic method).

#### 2.2.2 Tampa scale for kinesiophobia (TSK) (11)

The TSK was employed to assess the level of kinesiophobia. The scale comprises 17 items, each rated on a 4-point Likert scale (1–4), corresponding to “strongly disagree,” “disagree,” “agree,” and “strongly agree.” Items 4, 8, 11, and 12 are reverse scored.

The total score ranges from 17 to 68, with scores ≥37 indicating the presence of kinesiophobia. Higher scores reflect greater fear of movement, higher perceived pain intensity, and a stronger tendency toward fear-avoidance behaviors.

The TSK has been widely validated across various clinical populations; its Cronbach's *α* coefficient is 0.883, and its test-retest reliability is 0.798 (12, 13).

#### 2.2.3 Visual analogue scale (VAS) (14)

The VAS was used to evaluate pain intensity. Patients rated their pain subjectively by marking a point along the scale. Pain severity was categorized as follows: 0 = no pain, 1–3 = mild pain, 4–6 = moderate pain, and 7–10 = severe pain.

#### 2.2.4 Postoperative pain self-management behavior questionnaire (PPSMB) (15)

The PPSMB, adapted by Xu et al., was utilized to assess postoperative pain self-management behaviors in surgical patients. The scale has demonstrated good reliability, validity, and applicability. It encompasses two dimensions: pain knowledge acquisition and pain self-management, comprising a total of 10 items, each rated on a 4-point Likert scale (1–4), representing “unable to perform at all,” “occasionally able to perform,” “generally able to perform,” and “fully able to perform.” The total score ranges from 10 to 40, with higher scores indicating better pain self-management ability.

#### 2.2.5 Self-rating scale of sleep (SRSS) (16)

The SRSS consists of 10 items, each scored on a 5-point scale (1–5), yielding a total score from 10 to 50. Higher scores indicate poorer sleep quality; a score of 10 suggests minimal or no sleep disturbance, whereas a score of 50 indicates severe sleep impairment. The scale has a Cronbach's *α* coefficient of 0.6418 and a validity coefficient of 0.5625.

### 2.3 Data collection

Data were collected using a questionnaire-based survey. The investigators were members of the research team who had received standardized training in administering the kinesiophobia-related questionnaires, including unified instructions, precautions, and scoring procedures. Demographic and clinical data were extracted from electronic medical records. The TSK, VAS, PPSMB, and SRSS assessments were completed within 24 h after surgery. Questionnaires were administered through one-on-one interviews, during which investigators provided clarification as needed to ensure completeness and accuracy. Patients with a TSK score ≥37 on postoperative day 1 were reassessed on day 3; those who remained ≥37 on day 3 were reassessed again on day 5; and those with persistent TSK ≥37 on day 5 underwent a final assessment on the day of discharge. All data collection procedures adhered strictly to confidentiality principles, and completed questionnaires were securely stored to ensure patient privacy.

### 2.4 Statistical analysis

Statistical analyses were performed using R software (version 4.4.0). Normally distributed continuous variables are presented as mean ± standard deviation (*x¯* ± *s*), with between-group comparisons conducted using independent-samples *t*-tests (two groups) or one-way analysis of variance (ANOVA) (multiple groups). Non-normally distributed continuous variables are presented as median (Q1, Q3), with comparisons made via the Mann–Whitney *U* test (two groups) or the Kruskal–Wallis *H* test (multiple groups). Categorical variables are expressed as frequencies and percentages [*n* (%)], and were compared using the chi-square (*χ*^2^) test or Fisher's exact test, as appropriate. Independent variables were screened using Least Absolute Shrinkage and Selection Operator (LASSO) regression, followed by multivariate logistic regression analysis to identify factors influencing the occurrence and persistence of postoperative kinesiophobia. Receiver operating characteristic (ROC) curves were constructed, and the area under the curve (AUC) was calculated to evaluate the predictive performance of individual parameters and their combined model; ROC curves were compared using the DeLong test. A significance level of *α* = 0.05 was set, and *P* < 0.05 was considered statistically significant.

## 3 Results

### 3.1 Incidence and outcomes of kinesiophobia in patients undergoing thoracoscopic lung surgery

**Figure 1:**
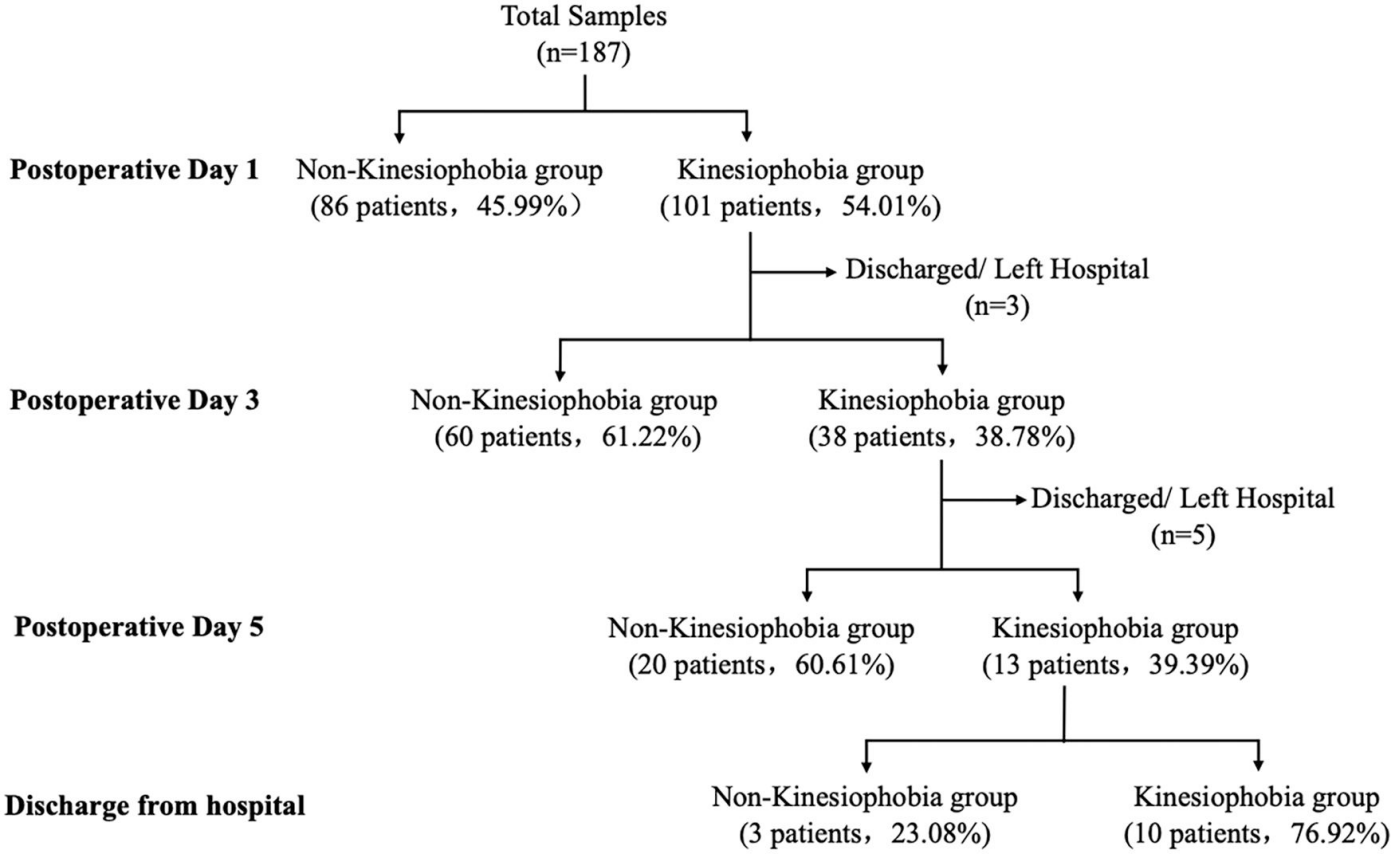
Incidence and progression of postoperative kinesiophobia among patients undergoing VATS lung surgery.

A total of 187 patients were ultimately included in the final analysis. Based on the TSK scores obtained on postoperative day 1, 101 patients (54.01%) were classified as having kinesiophobia, while 86 patients (45.99%) were classified as non-kinesiophobic. Patients with kinesiophobia were followed up every two days until discharge. Among the 187 patients, 86 (45.99%) remained non-kinesiophobic throughout the study period; 83 patients (44.39%) transitioned from kinesiophobia to non-kinesiophobia during follow-up; and the remaining 18 patients (9.63%) continued to experience kinesiophobia at the time of discharge. The incidence and progression of kinesiophobia at each follow-up time point are presented in Figure 1.

### 3.2 Comparison of scale scores between kinesiophobia and non-kinesiophobia groups at different time points

On postoperative day 1, the median TSK score in the kinesiophobia group was 41.00, with median VAS, PPSMB, and SRSS scores of 3.00, 22.00, and 24.00, respectively. In the non-kinesiophobia group, the median TSK score was 33.50, with median VAS, PPSMB, and SRSS scores of 3.00, 24.00, and 19.00, respectively. Compared with the non-kinesiophobia group, the kinesiophobia group exhibited higher VAS and SRSS scores and a lower PPSMB score. With respect to longitudinal changes, among patients who remained persistently kinesiophobic, the median TSK scores on postoperative day 3, day 5, and at discharge were 39.00, 41.00, and 38.00, respectively; the corresponding median VAS scores were 3.00, 2.00, and 1.50, indicating a gradual decline in pain intensity; median PPSMB scores were 27.00, 27.00, and 25.50; and median SRSS scores were 20.50, 19.00, and 20.50. In patients who transitioned from kinesiophobia to non-kinesiophobia, the median TSK scores at subsequent follow-up time points (day 3, day 5, and discharge) were 34.00, 35.00, and 36.00, respectively, showing a slight upward trend over time; median VAS scores were 2.00, 2.00, and 1.50, indicating a decreasing pain trajectory; PPSMB scores were 27.00, 29.50, and 33.50, reflecting progressive improvement in pain self-management ability; and SRSS scores were 25.00, 20.50, and 22.50. Detailed results are presented in Table 1.

**Table 1:** Scale scores of the study participants.

Scale score	Non-kinesiophobia group		Kinesiophobia group	
	M (Q1, Q3)	*n*_1_	M (Q1, Q3)	*n*_2_
TSK score				
T1	33.50 (32.00, 35.00)	86	41.00 (38.00, 42.00)	101
T2	34.00 (34.00, 36.00)	60	39.00 (38.00, 41.00)	41
T3	35.00 (32.50, 35.00)	20	41.00 (38.00, 41.00)	21
T4	36.00 (36.00, 36.00)	3	38.00 (37.00, 41.00)	18
VAS score				
T1	3.00 (2.00, 3.00)	86	3.00 (3.00, 4.00)	101
T2	2.00 (2.00, 3.00)	60	3.00 (2.00, 3.00)	41
T3	2.00 (2.00, 3.00)	20	2.00 (2.00, 3.00)	21
T4	1.50 (1.25, 1.75)	3	1.50 (1.00, 2.00)	18
PPSMB score				
T1	24.00 (21.25, 29.00)	86	22.00 (18.00, 25.00)	101
T2	27.00 (24.00, 31.00)	60	27.00 (24.00, 29.00)	41
T3	29.50 (27.00, 31.00)	20	27.00 (22.00, 30.00)	21
T4	33.50 (32.25, 34.75)	3	25.50 (22.00, 30.75)	18
SRSS score				
T1	19.00 (18.00, 25.00)	86	24.00 (19.00, 29.00)	101
T2	25.00 (19.00, 28.00)	60	20.50 (18.00, 27.75)	41
T3	20.50 (18.00, 27.50)	20	19.00 (16.00, 24.00)	21
T4	22.50 (18.75, 26.25)	3	20.50 (19.00, 28.00)	18

T1, postoperative day 1; T2, postoperative day 3; T3, postoperative day 5; T4, at discharge.

### 3.3 Factors influencing the occurrence of kinesiophobia on postoperative day 1

Based on TSK scores on postoperative day 1, patients were divided into a kinesiophobia group (TSK ≥ 37, *n* = 101, 54.01%) and a non-kinesiophobia group (TSK < 37, *n* = 86, 45.99%). Operative duration was significantly longer in the kinesiophobia group than in the non-kinesiophobia group (*Z* = −3.412, *P* < 0.001). In addition, VAS and SRSS scores were significantly higher, and PPSMB scores significantly lower, in the kinesiophobia group compared with the non-kinesiophobia group, with statistically significant differences for VAS (*Z* = −3.873, *P* < 0.001), PPSMB (*Z* = −3.303, *P* < 0.001), and SRSS (*Z* = −3.116, *P* = 0.001). No statistically significant differences were observed between the two groups regarding occupation, education level, smoking history, BMI, disease diagnosis, chest drainage tube size, or analgesia method. However, given that the Cronbach's *α* of the SRSS was only marginally acceptable, and its association did not reach statistical significance in the multivariate analysis, the potential impact of sleep on kinesiophobia warrants further investigation using more refined assessment tools and larger sample sizes. Detailed results are presented in Table 2.

**Table 2:** Comparison between kinesiophobia and non-kinesiophobia groups.

Variables	Total (*n* = 187)	Non-kinesiophobia group (*n* = 86)	Kinesiophobia group (*n* = 101)	Statistic	*P*
Gender (%)					
Male	82 (43.85)	34 (39.54)	48 (47.53)	0.902	0.342
Female	105 (56.15)	52 (60.46)	53 (52.47)		
Age (year)^a^	57.41 ± 12.57	57.07 ± 12.46	57.70 ± 12.72	−0.343	0.732
Occupation (%)^c^					
Worker	24 (12.83)	10 (11.63)	14 (13.86)	–	0.544
Individual	20 (10.70)	12 (13.95)	8 (7.92)		
Tutor	5 (2.67)	3 (3.49)	2 (1.98)		
Peasant	44 (23.53)	20 (23.26)	24 (23.76)		
Retiree	61 (32.62)	30 (34.88)	31 (30.69)		
Student	2 (1.07)	1 (1.16)	1 (0.99)		
Others	31 (16.58)	10 (11.63)	21 (20.79)		
Marital status (%)					
Married	170 (90.91)	77 (89.54)	93 (92.08)	0.121	0.728
Single/Divorced/Widowed	17 (0.09)	9 (10.46)	8 (7.92)		
Education level (%)					
Primary school and below	86 (45.99)	24 (27.91)	36 (35.64)	1.425	0.490
Junior high/Technical Secondary/Senior high	86 (45.99)	43 (50.00)	43 (42.57)		
College/Bachelor's degree	41 (21.93)	19 (22.09)	22 (21.78)		
Medical insurance coverage (%)					
Employee medical insurance	101 (54.01)	50 (58.14)	51 (50.49)	1.204	0.547
Resident medical insurance	82 (43.85)	34 (39.54)	48 (47.53)		
Other	4 (2.14)	2 (2.33)	2 (1.98)		
Residence status (%)^c^					
Living with Family	183 (97.86)	84 (97.67)	99 (98.02)	–	0.999
Living alone	4 (2.14)	2 (2.33)	2 (1.98)		
Smoking history (%)					
None	133 (71.12)	62 (72.09)	71 (70.30)	1.268	0.530
5–20 years	10 (5.35)	6 (6.98)	4 (3.96)		
20 years and above	44 (23.53)	18 (20.93)	26 (25.74)		
BMI (kg/m^2^)^a^	23.84 ± 3.11	23.93 ± 2.83	23.77 ± 3.35	0.354	0.723
Complications (%)					
No	82 (43.85)	40 (46.51)	42 (41.58)	0.279	0.597
Yes	105 (56.15)	46 (53.49)	59 (58.42)		
Operation duration (minutes)^b^	115.0 (74.50, 175.00)	92.50 (70.00, 153.00)	140.00 (90.00, 190.00)	−3.412	<0.001
Disease diagnosis (%)					
Benign	128 (68.45)	61 (70.93)	67 (66.34)	0.266	0.606
Malignant	59 (31.55)	25 (29.07)	34 (33.66)		
Chest drainage tube size (%)					
8	36 (19.25)	20 (23.26)	16 (15.84)	7.792	0.099
10	26 (13.90)	8 (9.30)	18 (17.82)		
12	29 (15.51)	15 (17.44)	14 (13.86)		
16	82 (43.85)	40 (46.51)	42 (41.58)		
24	14 (7.49)	3 (3.49)	11 (10.89)		
Analgesia method (%)^c^					
Analgesic pump	183 (97.86)	86 (100.00)	97 (96.04)	–	0.126
Other	4 (2.14)	0 (0.00)	4 (3.96)		
VAS score^b^	3.00 (3.00, 3.00)	3.00 (2.00, 3.00)	3.00 (3.00, 4.00)	−3.873	<0.001
PPSMB score^b^	22.00 (20.00, 27.00)	24.00 (21.25, 29.00)	22.00 (18.00, 25.00)	−3.303	<0.001
SRSS score^b^	23.00 (18.50, 27.00)	19.00 (18.00, 25.00)	24.00 (19.00, 29.00)	−3.116	0.001

a Continuous variables with normal distribution are expressed as mean ± standard deviation, with *t*-values reported.

b Continuous variables with non-normal distribution are expressed as median (interquartile range), with *Z*-values reported.

c Fisher's exact test.

### 3.4 Multivariate logistic regression analysis of factors influencing kinesiophobia on postoperative day 1

The occurrence of kinesiophobia on postoperative day 1 (yes = 1, no = 0) was set as the dependent variable. Variables with *P* < 0.05 in the univariate analysis (operative duration, VAS score, PPSMB score, and SRSS score) were entered in the multivariate logistic regression model. Multicollinearity was assessed using the variance inflation factor (VIF), and all variables showed VIF values <1.5 (operative duration: 1.02; VAS score: 1.00; PPSMB score: 1.08; SRSS score: 1.08), indicating no significant multicollinearity. The multivariate analysis demonstrated that operative duration (OR = 1.007, 95% CI: 1.002–1.013, *P* = 0.005) and VAS score (OR = 1.956, 95% CI: 1.360–2.921, *P* < 0.001) were independent risk factors for postoperative kinesiophobia, whereas the PPSMB score was an independent protective factor (OR = 0.926, 95% CI: 0.867–0.986, *P* = 0.019). The SRSS score was not statistically significant in the multivariate model (*P* = 0.119). Detailed results are presented in Table 3.

**Table 3:** Multivariate logistic regression analysis of factors influencing kinesiophobia on postoperative day 1.

Variables	*β*	S.E	Wald *χ*^2^	OR (95%CI)	*P*
Operation duration	0.007	0.002	2.795	1.007 (1.002, 1.013)	0.005
VAS score	0.670	0.193	3.464	1.956 (1.360, 2.921)	<0.001
PPSMB score	−0.076	0.032	−2.342	0.926 (0.867, 0.986)	0.019
SRSS score	0.043	0.027	1.558	1.044 (0.989, 1.104)	0.119

S.E., standard error; OR, odds ratio; CI, confidence interval.

### 3.5 ROC curve analysis of factors influencing kinesiophobia on postoperative day 1

**Figure 2:**
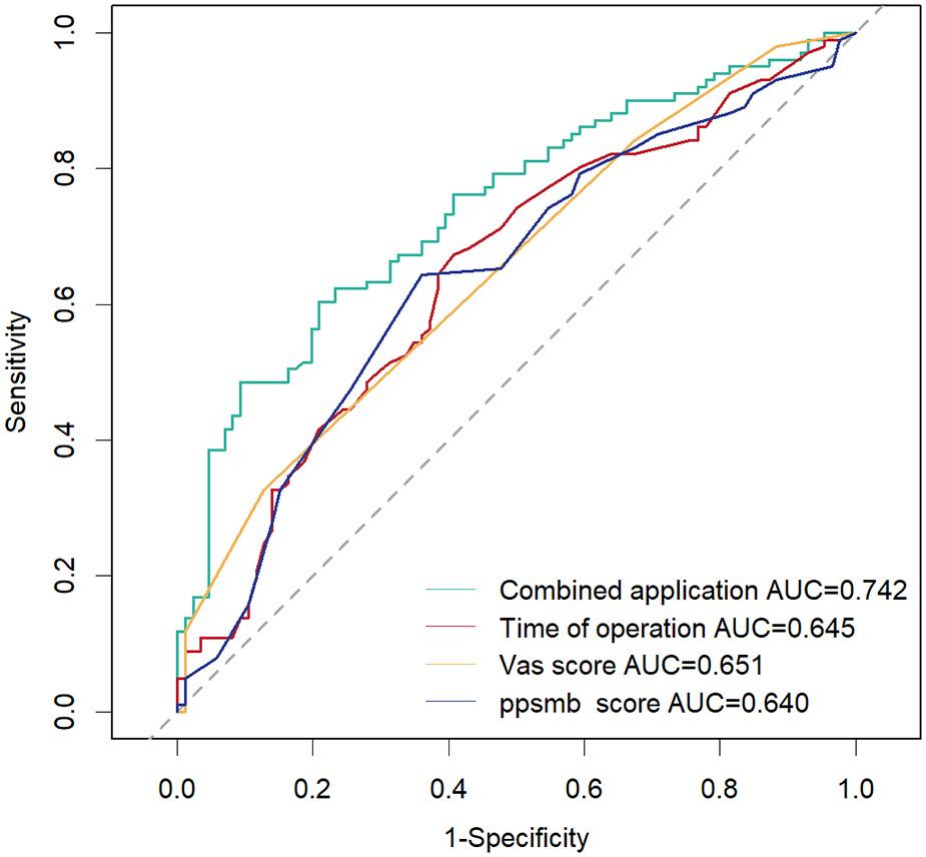
ROC curves of individual and combined parameters for predicting postoperative kinesiophobia.

Receiver operating characteristic (ROC) curves were constructed using operative time, VAS score, and PPSMB score as test variables, with the occurrence of postoperative kinesiophobia as the outcome variable. The optimal cut-off values for each indicator were determined using the maximum Youden index. The ROC analysis showed that the area under the curve (AUC) for operative duration, VAS score, and PPSMB score in predicting postoperative kinesiophobia were 0.645, 0.651, and 0.640, respectively. The optimal cut-off values were 107.50 min, 3.50, and 22.50, with corresponding sensitivities of 67.32%, 32.67%, and 64.35%, and specificities of 59.30%, 87.21%, and 63.95%, respectively. The combined application of these three parameters yielded an AUC of 0.742, with a sensitivity of 60.39% and a specificity of 79.07%. The combined model demonstrated a significantly higher AUC compared with each individual parameter alone (all *P* < 0.05). Detailed results are shown in Table 4 and Figure 2.

**Table 4:** ROC curve analysis of individual and combined parameters for predicting postoperative kinesiophobia.

Variables	AUC (95%CI)	Cut-off value	Sensitivity (%)	Specificity (%)	*Z* value	*P* value
Combined application	0.742 (0.671, 0.812)	–	60.39	79.07	–	–
Operation duration	0.645 (0.565, 0.724)	107.50	67.32	59.30	2.455	0.014
VAS score	0.651 (0.580, 0.722)	3.50	32.67	87.21	2.889	0.004
PPSMB score	0.640 (0.560, 0.720)	22.50	64.35	63.95	2.525	0.011

AUC, area under the curve; CI, confidence interval; *Z* and *P* values indicate comparisons between single predictors and the combined model.

### 3.6 Comparison among different kinesiophobia outcome groups from postoperative period to discharge

Based on TSK scores on postoperative day 1 and the three follow-up assessments, patients were categorized into three groups: non-kinesiophobia group (*n* = 86, 45.99%), the transition group (kinesiophobia to non-kinesiophobia, *n* = 83, 44.39%), and persistent kinesiophobia group (*n* = 18, 9.63%). Comparisons of baseline characteristics, disease-related factors, and surgical variables among the three groups revealed statistically significant differences in postoperative length of hospital stay, operative duration, postoperative pain severity, catheterization duration, VAS score, PPSMB score, and SRSS score (all *P* < 0.05). Detailed comparisons among the three groups are presented in Table 5.

**Table 5:** Comparison among three kinesiophobia outcome groups.

Variables	Non-kinesiophobia group (*n* = 86)	Kinesiophobia-to-non-kinesiophobia group (*n* = 83)	Kinesiophobia group (*n* = 18)	Statistic	*P*
Postoperative length of hospital stay (days)^b^	5.00 (4.00, 7.00)	6.00 (5.00, 8.00)	5.50 (5.00, 7.50)	21.444	<0.001
Operation duration (minutes)^b^	92.50 (70.00, 153.00)	140.00 (87.50, 192.50)	135.00 (102.50, 188.80)	11.896	0.002
Postoperative pain severity (%)					
Mild	73 (84.88)	47 (56.63)	9 (50.00)	19.116	<0.001
Moderate	13 (15.12)	36 (43.37)	9 (50.00)		
Duration of catheterization (days)^b^	3.00 (2.00, 3.00)	3.00 (2.5, 5.00)	3.00 (2.00, 3.75)	13.210	0.001
VAS score^b^	3.00 (2.00, 3.00)	3.00 (3.00, 4.00)	3.00 (3.00, 4.00)	15.118	<0.001
PPSMB score^b^	24.00 (21.25, 29.00)	22.00 (18.00, 25.50)	21.50 (18.25, 24.00)	11.030	0.004
SRSS score^b^	19.00 (18.00, 25.00)	25.00 (19.00, 29.00)	20.50 (19.00, 29.50)	10.210	0.006

b Continuous variables with non-normal distribution are expressed as median (interquartile range), with *Z*-values reported.

### 3.7 LASSO regression analysis of factors influencing different kinesiophobia outcomes from the postoperative period to discharge

Given the limited sample size and the relatively large number of independent variables, LASSO regression was employed to screen variables while simultaneously addressing multicollinearity. Categorical variables were entered into the LASSO regression model as dummy variables. The analysis identified four key variables—operative duration, VAS score, PPSMB score, and SRSS score—with an events-per-variable (EPV) ratio of 4.5. The LASSO coefficient convergence path and cross-validation plots are provided in the Supplementary Figures S1, S2.

### 3.8 Multinomial logistic regression analysis of factors influencing kinesiophobia outcomes

The kinesiophobia outcome category (non-kinesiophobia = 0, transition = 1, persistent = 2) was set as the dependent variable, and the four variables selected by LASSO regression were entered as independent variables in a multinomial logistic regression model, with the non-kinesiophobia group serving as the reference. Compared with the non-kinesiophobia group, operative duration (OR = 1.007, 95% CI: 1.002–1.013) was a significant risk factor for the transition group. Higher VAS scores were risk factors for both the transition group (OR = 1.978, 95% CI: 1.334–2.933) and the persistent kinesiophobia group (OR = 1.866, 95% CI: 1.026–3.395), whereas higher PPSMB scores were protective factors for both the transition group (OR = 0.934, 95% CI: 0.874–0.998) and the persistent kinesiophobia group (OR = 0.891, 95% CI: 0.797–0.997). Detailed results are presented in Table 6.

**Table 6:** Multinomial logistic regression analysis of factors influencing kinesiophobia outcomes.

Variables	Transition group (kinesiophobia to non-kinesiophobia)					Kinesiophobia group				
	*β*	S.E	Wald *χ*^2^	OR (95% CI)	*P*	*β*	S.E	Wald *χ*^2^	OR (95% CI)	*P*
Operation duration	0.007	0.002	2.691	1.007 (1.002, 1.013)	0.007	0.008	0.004	1.938	1.008 (0.990, 1.016)	0.053
VAS score	0.394	0.245	3.396	1.978 (1.334, 2.933)	0.001	0.111	0.343	2.043	1.866 (1.026, 3.395)	0.041
PPSMB score	−0.077	0.036	−2.018	0.934 (0.874, 0.998)	0.044	−0.153	0.062	−2.017	0.891 (0.797, 0.997)	0.044
SRSS score	0.058	0.029	1.749	1.052 (0.994, 1.113)	0.080	0.016	0.049	0.872	1.008 (0.916, 1.108)	0.872

The non-kinesiophobia group was used as the reference category; OR, odds ratio; CI, confidence interval.

## 4 Discussion

### 4.1 Current status of postoperative kinesiophobia in patients undergoing thoracoscopic lung surgery

This study revealed that on postoperative day 1, 86 patients (45.99%) were classified into the non-kinesiophobia group (TSK < 37), whereas 101 patients (54.01%) met the criteria for kinesiophobia (TSK ≥ 37), indicating a relatively high incidence of this condition. The median TSK score was 41.00 (interquartile range: 38.00, 42.00). These findings are consistent with those reported by Zhang et al. (17), who observed a mean TSK score of 40.18 ± 14.51 in patients undergoing thoracoscopic cancer surgery, suggesting that postoperative kinesiophobia is common and generally reaches a moderate-to-high level in this population. Notably, unlike previous studies, the present investigation incorporated continuous longitudinal follow-up of patients with TSK ≥ 37 on postoperative day 1. Among the initial kinesiophobia group, 83 patients (82.18%) transitioned to non-kinesiophobia by postoperative day 3 or day 5, whereas 18 patients (17.82%) remained persistently kinesiophobic until discharge. Importantly, the recovery rate from kinesiophobia dropped to 23.08% by postoperative day 5, with a non-recovery rate as high as 76.92%, indicating that the longer kinesiophobia persists, the more challenging recovery becomes.

The fear-avoidance model (FAM), proposed by Lethem et al. in 1983 (18), offers a theoretical framework to explain why some injuries heal uneventfully while others progress from acute to chronic conditions. According to this model, patients may perceive pain as a threatening stimulus and amplify pain-related information, thereby fostering fear and subsequent avoidance of movement. In 2012, Wen Hu (19) introduced the concept of “kinesiophobia” into the Chinese medical literature. Patients undergoing surgery are subjected to both disease-related and procedure-related physical and psychological stressors, which may precipitate avoidance behaviors. Pain further exacerbates psychological distress, resulting in excessive fear and avoidance of physical activity. Consequently, the occurrence and progression of postoperative kinesiophobia merit significant clinical attention.

### 4.2 Analysis of postoperative pain as a contributing factor

In the present study, the median VAS pain scores in the kinesiophobia group on postoperative day 1, day 3, day 5, and at discharge were 3.00 (3.00–4.00), 3.00 (2.00–3.00), 2.00 (2.00–3.00), and 1.50 (1.00–2.00), respectively, demonstrating a gradual downward trajectory. This suggests that reductions in pain intensity are associated with concurrent improvements in kinesiophobia. Moreover, pain scores in the kinesiophobia group were significantly higher than those in the non-kinesiophobia group (*Z* = −3.873, *P* < 0.001). Oskay (20) similarly reported that pain is a primary driver of kinesiophobia, with higher perceived pain levels associated with greater fear of movement.

ROC curve analysis identified a VAS cut-off value of 3.50, indicating that moderate-or-higher pain intensity serves as a significant predictors of postoperative kinesiophobia following thoracoscopic lung surgery, with a sensitivity of 32.67% and a specificity of 87.21%. The high specificity underscores pain as a robust positive predictor, whereas the relatively low sensitivity may be attributed to the inherently subjective nature of pain self-assessment, which can introduce misclassification. Therefore, clinicians should carefully evaluate patients’ ability to accurately self-report pain and supplement subjective ratings with clinical observation. Multidisciplinary perioperative pain management strategies can effectively reduce postoperative pain, attenuate stress responses, stabilize vital signs, improve emotional well-being, and decrease complications (21). Furthermore, time-point-matched preventive analgesia, tailored to individual pain trajectories, has been shown to effectively reduce moderate-to-severe postoperative pain and enhance recovery quality and patient satisfaction (22).

### 4.3 Analysis of postoperative pain self-management behavior

Our results demonstrated that PPSMB scores ranged from 24 to 33.5 in the non-kinesiophobia group and from 22 to 27 in the kinesiophobia group, with a statistically significant difference between the two groups (*P* = 0.04). Compared with the non-kinesiophobia group, the PPSMB score was a significant factor influencing the transition from kinesiophobia to non-kinesiophobia. Higher PPSMB scores (OR = 0.925, 95% CI: 0.862–0.993) were identified as a protective factor for recovery. Smith et al. (23) emphasized the critical importance of enhancing patients' postoperative pain self-management capabilities. Patients undergoing thoracic surgery often exhibit insufficient pain self-management skills, particularly those who are older, have lower educational attainment, or have not received prior pain education. It is essential to strengthen patient education regarding postoperative pain management, including knowledge of analgesic modalities, correct use of analgesics and patient-controlled analgesia devices, and overall pain awareness. Encouraging patients to actively engage in their own pain management can significantly improve analgesic efficacy. Hence, healthcare providers should focus on enhancing patients' abilities in pain self-assessment, knowledge acquisition, medication adherence, and utilization of non-pharmacological pain relief methods, all of which facilitate smoother postoperative recovery.

### 4.4 Analysis of operation duration

This study demonstrated that an operative duration ≥140 min was associated with an increased likelihood of kinesiophobia. Consistent with our findings, Liu et al. (24) reported a mean operative time of 144.9 min and showed that prolonged surgical duration is correlated with a more pronounced perioperative stress response. Extended operative times may exacerbate postoperative symptoms, thereby heightening the risk of kinesiophobia. Accordingly, operative duration may serve as an important predictor of postoperative kinesiophobia. The implementation of ERAS principles and thorough preoperative planning are crucial in this regard. Preoperative three-dimensional reconstruction of bronchial and vascular anatomy can reduce intraoperative bleeding and alleviate postoperative cough and pain. Additionally, advancements in surgical techniques and anesthetic management may differentially influence postoperative pain and kinesiophobia. Therefore, healthcare providers should reinforce their knowledge and application of ERAS protocols, strengthen patient education, and improve patients' understanding of their condition and self-care abilities, thereby reducing kinesiophobia and optimizing treatment outcomes.

### 4.5 Analysis of sleep status

This study showed that SRSS scores on postoperative day 1 were significantly associated with the occurrence of kinesiophobia in univariate analysis; however, sleep quality was not identified as an independent factor in the multivariate analysis. Zhang et al. (25) found that the most common postoperative symptoms included pain (100%), fatigue (98.64%), dyspnea (98.37%), cough (98.09%), and sleep disturbances (96.73%). Therefore, early attention to sleep status—either preoperatively or on postoperative day 1—may have greater clinical significance. It should also be noted that the SRSS evaluates sleep quality over the preceding month, which may limit its sensitivity in capturing acute postoperative changes.

### 4.6 Analysis of length of hospital stay

Multivariate logistic regression analysis comparing the transition group and the persistent kinesiophobia group against the non-kinesiophobia reference group revealed that a longer postoperative hospital stay (OR = 1.430, 95% CI: 1.052–1.946) was a risk factor for kinesiophobia. From postoperative day 3 to discharge, the recovery rate from kinesiophobia declined from 61.22% to 23.08%, indicating that the likelihood of recovery diminishes substantially over time. Conversely, kinesiophobia itself may impede recovery and prolong hospitalization. Patients in the persistent kinesiophobia group experienced extended hospital stays, increased financial burden, and impaired postoperative rehabilitation due to their fear of movement. Thus, early identification and timely intervention are imperative to improve clinical outcomes.

### 4.7 Limitations

This study has several limitations that should be acknowledged. First, although a longitudinal design was employed, the investigation was conducted at a single center with a relatively modest sample size, and follow-up was limited to the discharge period without assessing long-term outcomes. Extended follow-up is warranted in future research. Second, the persistent kinesiophobia group comprised only 18 patients, whereas the multivariable regression model included multiple predictors, yielding an EPV ratio of approximately 2.6, which falls below the recommended minimum threshold (typically ≥ 10). Consequently, the regression coefficients may be subject to overfitting, and the results should be interpreted with caution. Larger sample sizes are needed to validate these findings. Finally, the Cronbach's *α* coefficient of the SRSS in this study was 0.642, slightly below the conventional acceptability threshold of 0.70. Therefore, the findings related to sleep should be considered exploratory, and future studies should employ more precise sleep assessment instruments, such as the Pittsburgh Sleep Quality Index or polysomnography, to confirm these results.

## 5 Conclusion

In summary, the incidence of postoperative kinesiophobia in patients undergoing VATS lung surgery is relatively high. Healthcare providers should pay particular attention to moderate-to-severe postoperative pain, patients' pain self-management behaviors, sleep status, operation duration, chest tube dwell time, and length of hospital stay. Given that prolonged kinesiophobia poses increasing challenges to recovery, early identification, timely intervention, enhanced patient education, and structured rehabilitation guidance are essential to reduce its occurrence and facilitate early recovery, ultimately improving postoperative outcomes. This study is limited by its single-center design and relatively small sample size. Additionally, the methodology relied primarily on quantitative questionnaires without incorporating qualitative interviews with patients or systematic assessments of healthcare providers' knowledge, attitudes, and practices regarding kinesiophobia. Future investigations should include larger, multicenter cohorts, incorporate qualitative explorations of patients' perceptions, and evaluate providers' perspectives multidimensionally, so as to further elucidate the mechanisms of kinesiophobia, facilitate early identification and targeted intervention, and ultimately enhance postoperative recovery.

## Data Availability

The datasets presented in this study can be found in online repositories. The names of the repository/repositories and accession number(s) can be found in the article/Supplementary Material.

## Supplementary material

Supplementary Figure S1 Convergence path diagram of LASSO regression coefficients.

Supplementary Figure S2 LASSO regression cross-validation plot.
