# Supplementary material for: Postoperative kinesiophobia in patients undergoing video-assisted thoracoscopic lung surgery: a longitudinal study
Source: Front Surg. 2026 Jul 23;13:1874007. doi: 10.3389/fsurg.2026.1874007 (PMC13443308; doi:10.3389/fsurg.2026.1874007)
Supplement: Supplementary file 1 [file Datasheet1.docx]

**Supplementary Materials Figure S1.Convergence path diagram of LASSO regression coefficients**

**Supplementary Materials Figure S2.LASSO Regression Cross-Validation Plot**
